# Gap initiation with 20.35 mm: an initiator integrating the Al/CuO_x_ multilayer film and traditional electronic plug to enhance the ignition ability

**DOI:** 10.1098/rsos.181686

**Published:** 2019-05-01

**Authors:** Debin Ni, Guoqiang Yu, Shengnan Shi, Dong Xu, Enyi Chu, Chunpei Yu, Zilong Zhen, Wenchao Zhang

**Affiliations:** 1 Science and Technology on Applied Physical Chemistry Laboratory, Shaanxi Applied Physics and Chemistry Research Institute, Xi'an 710061, People's Republic of China; 2 School of Chemical Engineering, Nanjing University of Science and Technology, Nanjing 210094, People's Republic of China

**Keywords:** Al/CuO, nanothermite, ignition ability, gap initiation

## Abstract

In order to enhance the ignition ability and reliability of traditional electronic initiators, a novel electronic initiator has been designed to integrate with a nanothermite multilayer film and an electrode plug. The Al/CuO_x_ nanothermite multilayer film with different thickness is deposited on the surface of the electrode plug by magnetron sputtering which uses Pt–W wire as electronic resistance. The exothermicity of Al/CuO_x_ nanothermite multilayer film is so favourable that the ignition ability of electronic initiator is significantly improved. The full firing-voltage sensitivity of the electronic initiator is 10.8 V. The thickness of Al/CuO_x_ multilayer film has negligible effects on the ignition time and ignition energy, but leads to great impacts on the function time, the maximum length of combustion flame and ignition ability. The electrical ignition experiments have exhibited outstanding ignition ability, since the electronic initiator can easily fire the insensitive ignition composition of boron-potassium nitrate (B-KNO_3_) tablet in a gap of 20.35 mm. It proves that this novel proposal of remoulding the traditional electronic ignition devices will distinctly improve the ignition ability and reliability of electronic initiator.

## 1. Introduction

Bridge wire is normally used in traditional electronic initiators to initiate the subsequent energy materials [1]. Because of its low cost, convenient fabrication and low firing energy, it has been extensively applied in numerous civilian and military applications such as airbags in automobiles [2], microsatellites, missiles [3], nuclear weapons [4], rockets [5] and many other ordnance systems [6]. Since the energy materials are loaded manually in traditional electronic initiators, their safety and reliability have faced a long-term dilemma. The appearance of semiconductor bridge (SCB) [7], which is fabricated with standard microsystem techniques to allow mass production, remarkably causes a progress in the security and reliability of initiator [8]. The SCB has been usually taken as an initiator in military, civil and aerospace fields, since it is of better instantaneity, higher safety and lower firing energy than those of conventional bridge wires [9,10]. Because the ignition ability of SCB is heavily dependent on the stimulation energy with microseconds of reaction, it may not be sufficient to provide a reliable ignition if there is a gap between SCB and primary contact energetic material. Furthermore, the primary contact energetic material within SCB must be composed of extraordinarily sensitive initiating compositions; otherwise, the safety of ignition system will not be guaranteed [11].

In order to improve the ignition ability of SCB, multilayered metastable intermolecular composites (MICs), which usually comprised the nanoscale fuels and reductants, such as Al/CuO [12–15], Al/MoO_3_ [16,17] and Al/NiO [18–20], have been introduced into the SCB. The multilayer reactive films have been adopted as heat sources in many areas, such as micro-actuators and micro-ignitions. The exothermic reaction of MICs can release a great amount of heat and hyperthermal products [21], which can be ejected several millimetres or more in distance; as a result, the energetic material can be easily fired even if there is no physical contact between the initiator and the energetic material [22]. At the same time, the MICs could be easily integrated with SCB using magnetron sputtering, which could avoid manual operation to keep the reliability of initiator. Although the ignition ability and reliability of SCB initiator have been improved, the firing voltage is usually more than dozens of volts, which does not apparently match with the smart and micro-ignition system. In addition, the fabrication process of SCB initiator is more expensive and time-consuming than that of traditional electrode plug. If the electronic initiator is constituted by the integration of the bridge wire electrode plug and a multilayered nanothermite film, not only will its ignition ability be improved, but also its reliability will be promoted. Hence, a multilayer thermite film of Al/CuO_x_ has been integrated with bridge wire electronic initiator as a novel initiator. The multilayered thermite film of Al/CuO_x_ was deposited in different thickness on the surface of the electrode plug, where Pt–W wire was used as resistance by magnetron sputtering. The morphology, phase and exothermicity of Al/CuO_x_ multilayered thermite film have been investigated by field emission scanning electron microscope (SEM, Hitachi, S-4800), energy-dispersive X-ray spectrum (EDS), X-ray diffraction (XRD, Bruker, D8 Advance) and differential scanning calorimetry (DSC, Netzsch, STA449C). Not only the ignition performances and firing-voltage sensitivity of the electronic initiator, but also the effect of its thickness on the ignition ability have been characterized and are discussed in detail.

## 2. Experimental section

### 2.1. Materials

Al and CuO sputter targets (7 cm in diameter, 6 mm in thickness) were purchased from Jiangxi Ketai Advanced Material Co. Ltd. Acetone and ethanol were bought from Shanghai Chemical reagent Co. Ltd, positive photoresist (BP212) was gained from Kempur Microelectronics Inc. The electrode plugs were fabricated industrially. Deionized water was used in all experiments in the study.

### 2.2. Fabrication of the multilayered film of Al/CuO_x_

**Figure 1.**
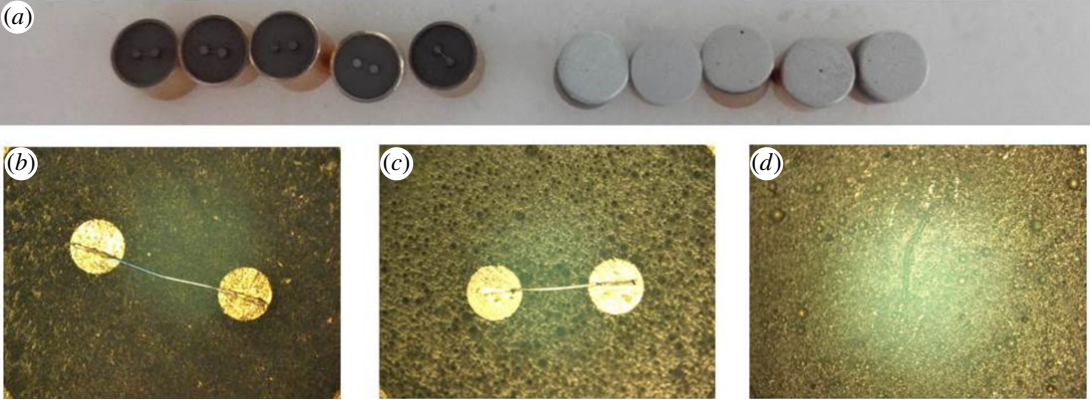
The initiators were deposited Al/CuO_x_ films (*a*). The microscope images of (*b*) without Al/CuO_x_ films, (*c*) with Al/CuO_x_ film, (*d*) after ignition electrode plug.

The Pt–W wire (8 µm in diameter, electric resistance 7 ± 0.2 Ω) was soldered on the electrode plug using stored energy welding. The electrode plug was cleaned ultrasonically with acetone, ethanol and deionized water in sequence for 30 min and dried in atmosphere before it was placed into the mould. The Al and CuO targets were sputtered at 250 W with a DC power source and at 200 W with an RF power source, while the ultra-high purity argon gas was selected as the working gas at a flow rate of 20 sccm (standard cubic centimetres per minute). All the deposition processes were controlled by a computer. The single layer thickness of the Al and CuO films, which were alternately deposited, were set to be 100 and 200 nm, respectively. The total thickness of Al/CuO_x_ multilayer film was 3.0, 4.5 and 8.0 µm, respectively. The images of electrode plugs without and with Al/CuO_x_ multilayer film are shown in figure 1.

## 3. Results and discussion

**Figure 2.**
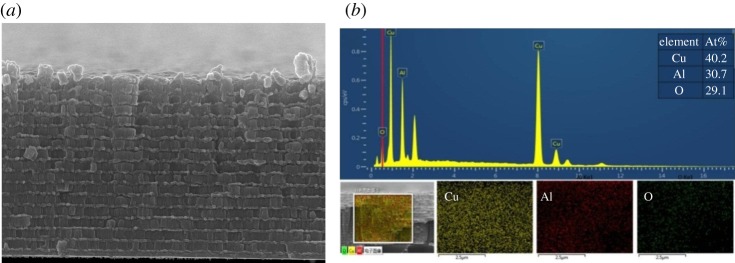
The cross-sectional SEM image (*a*), the EDS and corresponding elemental mappings (*b*) of the deposited Al/CuO film.

SEM was used to observe the cross-section of the multilayered Al/CuO_x_ film. The distinct layered structure of Al/CuO_x_ multilayer film with the thickness of 4.5 µm is shown in figure 2, and the images of Al/CuO_x_ multilayer film with the thickness of 3.0 and 8.0 µm are shown in electronic supplementary material, figure SI 1. EDS was used to estimate the elemental distributions by integration of peak areas, as shown in figure 2*b*. Three elements, Al, O and Cu, appeared in the EDS spectra of the Al/CuO_x_ multilayer film with the corresponding SEM image. The atomic percentages (At%) of these elements were 40.2, 30.7 and 29.1, respectively. The molar ratio between Al and CuO was (about 0.51) lower than the theoretical value (0.67).

**Figure 3.**
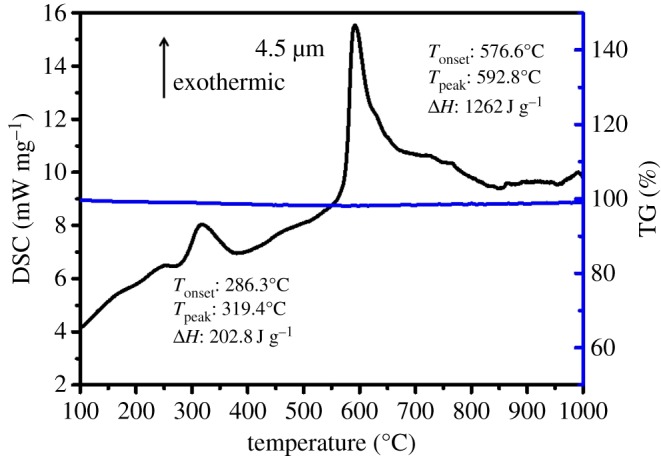
The DSC and TG plots of the multilayer Al/CuO_x_ film with 4.5 µm.

DSC was used to investigate the thermography of the multilayered Al/CuO_x_ film. The sample for DSC measurement was fabricated as follows: first, the multilayered film was deposited on the silicon substance with positive photoresist; second, the silicon plate was immersed into the solvent of acetone to remove photoresist three times; finally, the plate solid was dried in oven at 30°C. The film was heated from room temperature to 1000°C at a heating rate of 20 K min^−1^ under ultra-pure nitrogen at a 20 ml min^−1^ flow. The DSC curve of Al/CuO_x_ multilayer film with 4.5 µm is displayed in figure 3. Two major exothermic peaks appeared during the entire thermite reaction. One weak exothermic peak was observed with the onset temperature around 286.3°C and a heat output of 202.8 J g^−1^ may be attributed to the recrystallization of the native Al–Cu–O interface or low-temperature oxidation–reduction reaction [23], which was not expected to play a main role in the ignition process. The other huge and sharp exothermic peak appeared with the onset temperature of around 576.6°C and a heat output of 1262 J g^−1^, which resulted from the solid–solid reaction between Al with CuO. The maximum exothermic peak at 592.8°C meant that the multilayered Al/CuO_x_ film had reacted prior to the melting point of Al. The total amount of the released heat was approximately 1464.8 J g^−1^ which was much lower than the theoretical value 4079 J g^−1^, but the ignition ability was still very strong. As shown in the TG curve in figure 3, the mass of the multilayered Al/CuO_x_ film barely changed during the entire reaction, which confirmed that the first exothermic peak was not attributed to the residual photoresist. In addition, the total heat release of Al/CuO_x_ multilayer film with the thickness of 3 and 8 µm was 1440 and 1548 J g^−1^ (electronic supplementary material, figure SI 2), respectively. This indicates that the thickness exhibits little impact on the heat release.

**Figure 4.**
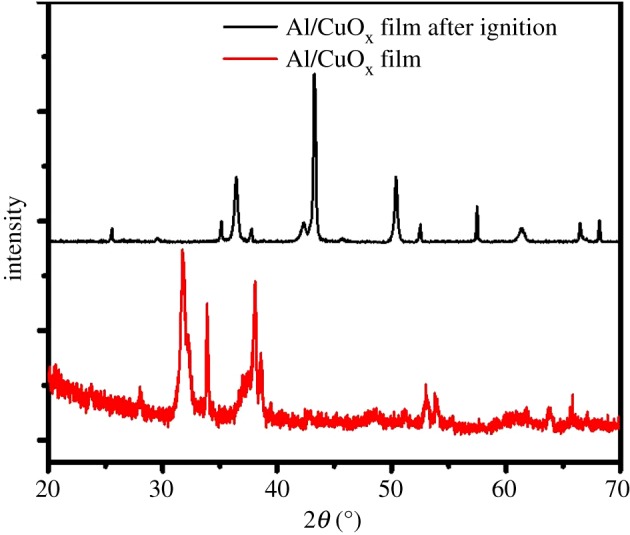
The XRD patterns of the multilayered Al/CuO_x_ film.

The XRD pattern of the Al/CuO_x_ multilayer film in figure 4 exhibits the diffraction peaks at 38.47°, 44.74°, 65.13° and 78.22° corresponding to crystal planes (111), (200), (220) and (311) of Al (JCPDS04-0787). The diffraction peaks at 35.64°, 36.34°, 58.31°, 63.93°, 65.03° and 75.51° were attributed to the crystal planes (111), (220), (311), (400), (511) and (440) of mineral paramelaconite (Cu_4_O_3_, JCPDS49-1830), respectively. This phase was a little copper richer than the CuO target material, which meant that some oxygen was lost during deposition [24]. The diffraction peaks at 43.29°, 50.43° and 74.13° were ascribed to the crystal planes (111), (200) and (220) of copper (Cu, JCPDS04-0836). The diffraction peaks at 29.63°, 36.50°, 42.40° and 61.52° were ascribed to the crystal planes (110), (111), (200) and (220) of copper (CuO, JCPDS65-3288), respectively. The CuO was decomposed from mineral paramelaconite, and Al was completely consumed during the reaction.

The firing-voltage sensitivity of the initiators was tested according to the Langlie method using the discharge of a 100 µF capacitor. The maximum firing-voltage (*V*_max_) and the minimum firing-voltage (*V*_min_) were assumed to be 20 and 0 V, respectively. After 18 times of experiments, the 50% and 99.9% firing-voltage sensitive was 8.41 and 10.80 V, respectively. The experimental data are summarized in table 1.

**Table 1.** The results from the firing-voltage sensitivity tests of initiators. *X*_max_: the maximum firing-voltage 20 V; *X*_min_: the minimum firing-voltage 0 V; *i*: test number; *X*_i_: charge voltage; *N*_i_: 1 (fire), 0 (unfire); *X*′_i_: next value of charge voltage.

*i*	1	2	3	4	5	6	7	8	9
*X*_i_ (V)	10	5	7.5	13.75	10.62	7.81	3.91	5.86	8.24
*N*_i_	1	0	0	1	1	1	0	0	0
*X*′_i_	*X*_min_	*X*_1_	*X*_max_	*X*_3_	*X*_2_	*X*_min_	*X*_6_	*X*_5_	*X*_4_

*i*	10	11	12	13	14	15	16	17	18
*X_i_* (V)	10.99	9.62	7.74	8.68	8.21	8.45	9.72	9.08	8.65
*N_i_*	1	1	0	1	0	0	1	1	1
*X*′*_i_*	*X*_9_	*X*_8_	*X*_11_	*X*_12_	*X*_13_	*X*_9_	*X*_15_	*X*_14_	

**Figure 5.**
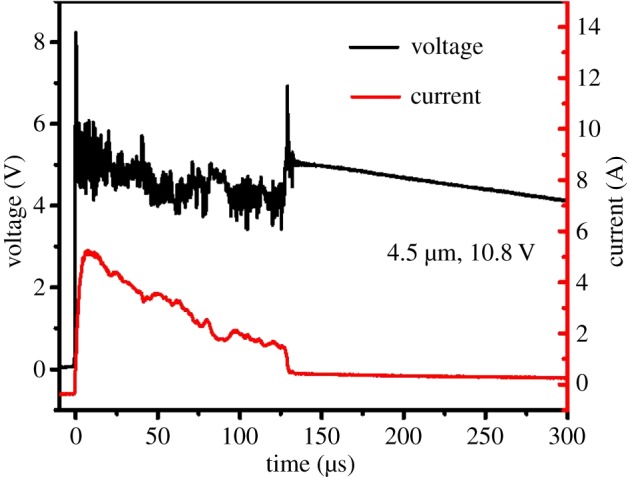
The voltage–current curve of initiator with 4.5 µm multilayered Al/CuO_x_ film.

In order to investigate the instantaneity and ignition energy of the initiators, the voltage and current of initiators during the process of ignition have been recorded using a high-speed digital storage oscilloscope. The ignition time was defined as the first maximum of the current peak, while the ignition energy was calculated by the formula $E=\int_{o}^{t}\mathrm{UIdt}$ [25]. The resulting data are listed in electronic supplementary material, table SI 1. The voltage–current curve for the initiator with the 4.5 µm multilayered Al/CuO_x_ film is exhibited in figure 5, and the other two curves are shown in electronic supplementary material, figure SI 3. The current instantly reached up to 4.93 A at 4.38 µs, where the voltage fluctuated around a relatively constant value of 5.0 V. The possible reason was that the unreacted Al and the product Cu were conductors, which irregularly splashed in the late process of discharge, to cause the fluctuation of voltage. The current was obviously decreased by the end of discharge. From the electronic supplementary material, table SI 1, the thickness of the multilayered Al/CuO_x_ film led to little effects on the ignition time and ignition energy, but great impacts on the function time. When the thickness of the multilayered Al/CuO_x_ film increased from 3.0 to 8.0 µm, the function time was increased from 83.95 to 207.60 µs, which contributed to the more and more reaction mass with increasing film thickness.

**Figure 6.**
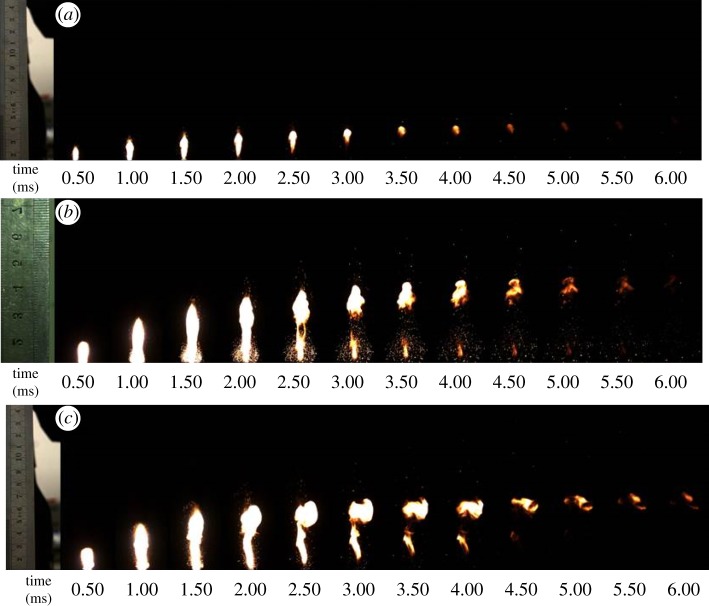
The high-speed camera images of initiators with the multilayered Al/CuO_x_ film of 3.0 µm (*a*), 4.5 µm (*b*) and 8.0 µm (*c*).

The combustion propagation processes were recorded by a high-speed camera (Redlake Motion Xtra HG-100 K) with 20 000 frames per second and the interval time of 0.25 ms for each frame image, as exhibited in figure 6. For all initiators with different thickness of the multilayered Al/CuO_x_ film, a small and bright flame flashed at 0.25 ms after ignition. The bright flame steadily grew and rapidly propagated forward. A great amount of hot solid particles erupted like a volcano at the same time. As the time went on, the brightness of flames got reduced. The videos of three kinds of initiators were recorded (see electronic supplementary material). The combustion durations of the multilayered Al/CuO_x_ film were 4.75, 5.75 and over 6.0 ms for 3.0, 4.5 and 8.0 µm film thickness, respectively. Among the images of three kinds of films, the area and the maximum length of the combustion flame was the smallest for the 3.0 µm film. The flame was spread over 4.5 cm in length for the initiator with 8.0 µm. The long and sustained combustion flame benefits the ignition ability of the electrode plug. The high-speed camera images of the electrode plug are shown in the electronic supplementary material, figure SI 4.

**Figure 7.**
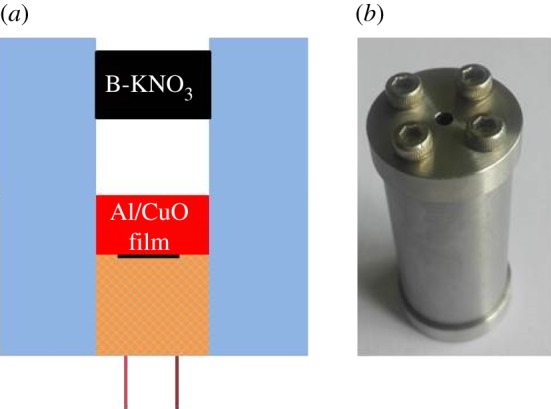
The gap test schematic drawing (*a*) and the photograph of device (*b*).

In order to investigate the ignition ability of initiators, electrical explosion experiments, in which a serial of gaps were designed, were carried out to fire the insensitivity ignition composition of B-KNO_3_ tablet with the density of 2 g cm^−3^, and the charge voltage was 10.8 V. The test set-up of ignition is shown in figure 7. Initially, the tablet closely contacted the multilayered Al/CuO_x_ film, if the B-KNO_3_ tablet was fired, the gap will be expanded until it could not be ignited. The results are shown in electronic supplementary material, table SI 2. Although these three initiators can easily fire the B-KNO_3_ tablet when both of them are kept in close contact, the ignition ability was extremely different. The initiation gap changed from 2.20 to 8.06 mm as the film thickness increased from 3 to 4.5 µm. Surprisingly, the initiation gap was actually enlarged to 20.35 mm for the 8.0 µm film, because the heat output distinctly increased with increasing thickness. The electrode plug without Al/CuO_x_ multilayer film was in close contact to ignite the B-KNO_3_ tablet, but the B-KNO_3_ tablet could not be fired even when the charge voltage was 50 V. The novel initiator is promising to be used in gap initiator system to enhance the safety and reliability of the traditional electropyrotechnic initiator.

## 4. Conclusion

The novel initiators, which were integrated at the traditional electrode plug and three different thicknesses of multilayered Al/CuO_x_ films, have been successfully deposited on the surface of the electrode plug using magnetron sputtering. The thickness has little effect on the ignition time and ignition energy, but great influence on the function time. Although the heat output of the multilayered Al/CuO_x_ film was relatively lower than the theoretical heat release, theses igniters can easily fire the insensitive ignition composition of the B-KNO_3_ tablet with a full firing-voltage of 10.8 V, even the gap between the electrode plug and the tablet was prolonged to be 20.35 mm for the 8.0 µm film. The super ignition ability was attributed to the long combustion frame and a great amount of erupted hot solid particles during the reaction. The excellent firing parameters and outstanding ignition ability of this kind of initiator can optimize the traditional electronic initiators remarkably, especially the non-contact initiation system. These initiators are promising in military and civilian applications.

## Supplementary Material

Reviewer comments

## Data accessibility

The electronic supplementary material of this article has been uploaded on the Dryad Digital Repository at: https://doi.org/10.5061/dryad.j5s606j [26].
