# Supplementary material for: Gap initiation with 20.35 mm: an initiator integrating the Al/CuOx multilayer film and traditional electronic plug to enhance the ignition ability
Source: R Soc Open Sci. 2019 May 1;6(5):181686. doi: 10.1098/rsos.181686 (PMC6549997; doi:10.1098/rsos.181686)
Supplement: Reviewer comments [file rsos181686_review_history.pdf]

THE ROYAL SOCIETY
PUBLISHING

ROYAL SOCIETY
OPEN SCIENCE

Gap initiation with 20.35 mm: an initiator integrating the Al/CuO_x multilayer film and traditional electronic plug to enhance the ignition ability

Debin Ni, Guoqiang Yu, Shengnan Shi, Dong Xu, Enyi Chu, Chunpei Yu, Zilong Zhen and Wenchao Zhang

Article citation details
R. Soc. open sci. **6**: 181686.
http://dx.doi.org/10.1098/rsos.181686

Review timeline

Original submission:	15 October 2018
1st revised submission:	1 March 2019
2nd revised submission:	4 April 2019
Final acceptance:	5 April 2019

Note: Reports are unedited and appear as submitted by the referee. The review history appears in chronological order.

Review History

RSOS-181686.R0 (Original submission)

Review form: Reviewer 1 (Yimin Chao)

Is the manuscript scientifically sound in its present form?
Yes

Are the interpretations and conclusions justified by the results?
Yes

Is the language acceptable?
No

Is it clear how to access all supporting data?
Yes

Reports © 2019 The Reviewers; Decision Letters © 2019 The Reviewers and Editors; Responses © 2019 The Reviewers, Editors and Authors. Published by the Royal Society under the terms of the Creative Commons Attribution License http://creativecommons.org/licenses/by/4.0/, which permits unrestricted use, provided the original author and source are credited

Do you have any ethical concerns with this paper?
No

Have you any concerns about statistical analyses in this paper?
No

Recommendation?
Accept with minor revision (please list in comments)

Comments to the Author(s)
The author have presented an interesting work on multilayer film of Al/Cux synthesized by a classical route. The work is plausible and useful. However, the major problem to me is the language really needs to be polished, with the following point to be considered:

1. replace the full stop with comma after plug in the second line in abstract;
2. page 2, line 4 in the second paragraph, double check Al/Ni, it might be Al/NiO;
3. page 5 line 7, replace weekly with weak;
4. page 5 line 16, the statement on thickness effect is incorrect;
5. page 6, line 4, replace was with were; a lot of grammar errors in this paragraph and beyond;

Review form: Reviewer 2

Is the manuscript scientifically sound in its present form?
Yes

Are the interpretations and conclusions justified by the results?
No

Is the language acceptable?
No

Is it clear how to access all supporting data?
Yes

Do you have any ethical concerns with this paper?
No

Have you any concerns about statistical analyses in this paper?
I do not feel qualified to assess the statistics

Recommendation?
Major revision is needed (please make suggestions in comments)

Comments to the Author(s)
Review Report for RSOS-181686

General Comments:
This manuscript reported on the preparation and performance characterization of an electronic initiator which is based on nanothermite multilayer film and electrode plug. The ignition ability of electronic initiator with different thickness of energetic film was investigated and proved to be

with an improved safety and reliability. The idea of integrating nanothermite multilayer film and electrode plug directly is interesting, simple and easy to implement. However, the reviewer has some concerns as stated in specific comments. These issues should be clarified before the manuscript could be considered for publication.

Specific Comments:
1. Section 3, the total thickness of nanothermite film was demonstrated to have little effect on heat release on page 5. However, page 9, authors stated the "heat output distinctly increased as the thickness raising", which seems to be a contradiction. Please explain. Besides, most papers show that the factor that affects the exothermic performance of multilayer energetic materials is the modulation period. But here, much better ignition ability in the gap test can be achieved by simply increasing the thickness of film. What's the possible mechanism? It would be better to add more in-depth discussion.
2. Introduction part, the authors used SCB as a comparative example. However, most of the cited references have no relation with SCB, e.g. [14], [17], [18], [20], [23-28] etc. Please check.
3. Figure 1, is it possible to add magnified figures of one electrode plug with and without nanothermite multilayer film, especially the area between electrodes, to see some details?
4. Section 2.2, the authors set the single layer thickness of Al film and CuO film were 100 nm and 200 nm, but in Figure 2a, the two layers seem not to be twice in thickness.
5. Figure 2b, why is there no obvious layering in EDS mapping? In addition, there are several unlabeled peaks in EDS plot.
6. Page 5, it seems the exothermic reaction after Al melting (660 C) contributes to heat release as well. Hence, the statement that "heat output was caused by solid-solid reaction" is not appropriate here.
7. Page 5, last sentence, the Figure SI 2 is voltage-current curve instead of DSC plots. Please check.
8. Page 6, the described Cu peaks (43.29, 50.43 and 74,13 degree) are not that obvious in the product curve of Figure 4. Besides, what do the sharp peaks in the product curve at around 53 and 55 degree stand for?
9. Page 7, please define function time. Because from Figure 6, the flame root of the 8 μ m-thick sample was extinguished earlier than that of 4.58 μ m-thick sample.
10. In order to highlight the advantages of Al/CuOX film, it is suggested to add the high-speed camera ignition images of traditional electronic plug (optional).
11. Page 9, "Although three kinds of initiators can easily fire the B-KNO3 tablet when it was intimately contacted with film, the ignition ability was much more differently." The authors did not provide related experimental data to support this sentence.
12. Page 9, last sentence, what does the "same condition" mean? Same contact or same gap distance?
13. Why the authors think the Al/CuOx layer can improve the safety of initiator? From this manuscript, the Al/ CuOx layer decreased the ignition voltage and it is possible to ignite even the gap increased to over 20mm. Doesn't it mean the initiator is less safe?
14. Two related papers should be included in the references: Journal of Materials Science 47, 1296-1305, 2012; ACS applied materials & interfaces 6, 10497-10505, 2014.
15. A carefully examine of the English grammar, sentence patterns and phrases of the entire manuscript sentence by sentence is sincerely recommended. E.g. the first sentence of Abstract part is completely unclear.

Decision letter (RSOS-181686.R0)

07-Feb-2019

Dear Dr Ni,

The editors assigned to your paper ("Gap initiation with 20.35 mm: an initiator integrating the Al/CuOx multilayer film and traditional electronic plug to enhance the ignition ability") have now received comments from reviewers. We would like you to revise your paper in accordance with the referee and Associate Editor suggestions which can be found below (not including confidential reports to the Editor). Please note this decision does not guarantee eventual acceptance.

Please submit a copy of your revised paper before 02-Mar-2019. Please note that the revision deadline will expire at 00.00am on this date. If we do not hear from you within this time then it will be assumed that the paper has been withdrawn. In exceptional circumstances, extensions may be possible if agreed with the Editorial Office in advance. We do not allow multiple rounds of revision so we urge you to make every effort to fully address all of the comments at this stage. If deemed necessary by the Editors, your manuscript will be sent back to one or more of the original reviewers for assessment. If the original reviewers are not available, we may invite new reviewers.

To revise your manuscript, log into http://mc.manuscriptcentral.com/rsos and enter your Author Centre, where you will find your manuscript title listed under "Manuscripts with Decisions." Under "Actions," click on "Create a Revision." Your manuscript number has been appended to denote a revision. Revise your manuscript and upload a new version through your Author Centre.

When submitting your revised manuscript, you must respond to the comments made by the referees and upload a file "Response to Referees" in "Section 6 - File Upload". Please use this to document how you have responded to the comments, and the adjustments you have made. In order to expedite the processing of the revised manuscript, please be as specific as possible in your response.

In addition to addressing all of the reviewers' and editor's comments please also ensure that your revised manuscript contains the following sections as appropriate before the reference list:

• Ethics statement (if applicable)
If your study uses humans or animals please include details of the ethical approval received, including the name of the committee that granted approval. For human studies please also detail whether informed consent was obtained. For field studies on animals please include details of all permissions, licences and/or approvals granted to carry out the fieldwork.

• Data accessibility
It is a condition of publication that all supporting data are made available either as supplementary information or preferably in a suitable permanent repository. The data accessibility section should state where the article's supporting data can be accessed. This section should also include details, where possible of where to access other relevant research materials such as statistical tools, protocols, software etc can be accessed. If the data have been deposited in an external repository this section should list the database, accession number and link to the DOI for all data from the article that have been made publicly available. Data sets that have been deposited in an external repository and have a DOI should also be appropriately cited in the manuscript and included in the reference list.

If you wish to submit your supporting data or code to Dryad (http://datadryad.org/), or modify your current submission to dryad, please use the following link:
http://datadryad.org/submit?journalID=RSOS&manu=RSOS-181686

• Competing interests
Please declare any financial or non-financial competing interests, or state that you have no competing interests.

• Authors' contributions
All submissions, other than those with a single author, must include an Authors' Contributions section which individually lists the specific contribution of each author. The list of Authors should meet all of the following criteria; 1) substantial contributions to conception and design, or acquisition of data, or analysis and interpretation of data; 2) drafting the article or revising it critically for important intellectual content; and 3) final approval of the version to be published.

All contributors who do not meet all of these criteria should be included in the acknowledgements.

We suggest the following format:
AB carried out the molecular lab work, participated in data analysis, carried out sequence alignments, participated in the design of the study and drafted the manuscript; CD carried out the statistical analyses; EF collected field data; GH conceived of the study, designed the study, coordinated the study and helped draft the manuscript. All authors gave final approval for publication.

• Acknowledgements
Please acknowledge anyone who contributed to the study but did not meet the authorship criteria.

• Funding statement
Please list the source of funding for each author.

Once again, thank you for submitting your manuscript to Royal Society Open Science and I look forward to receiving your revision. If you have any questions at all, please do not hesitate to get in touch.

Kind regards,
Royal Society Open Science Editorial Office
Royal Society Open Science
openscience@royalsociety.org

on behalf of Professor R. Kerry Rowe (Subject Editor)
openscience@royalsociety.org

Associate Editor's comments:
Thank you for the submission to Royal Society Open Science. We have now received two reviewers' reports on your paper. Given the numerous concerns of referee 2, we require you to complete a revision of your paper that addresses these before we can consider the paper further for publication. You should incorporate their recommendations in full or provide a scientifically valid rebuttal of those concerns in your revision -- if you do not do so, and your changes or responses do not satisfy the reviewer, you may not receive a second opportunity to address these

concerns. Furthermore, the more critical reviewer indicates the manuscript has substantial linguistic errors. Please ensure that you utilise a language polishing service (for instance https://royalsociety.org/journals/authors/language-polishing/) before resubmitting, and ensure that you include evidence of their support in your revision -- if you do not do so, the paper will be returned to you. Good luck!

Comments to Author:

Reviewers' Comments to Author:
Reviewer: 1

Comments to the Author(s)
The author have presented an interesting work on multilayer film of Al/Cux synthesized by a classical route. The work is plausible and useful. However, the major problem to me is the language really needs to be polished, with the following point to be considered:

1. replace the full stop with comma after plug in the second line in abstract;
2. page 2, line 4 in the second paragraph, double check Al/Ni, it might be Al/NiO;
3. page 5 line 7, replace weekly with weak;
4. page 5 line 16, the statement on thickness effect is incorrect;
5. page 6, line 4, replace was with were; a lot of grammar errors in this paragraph and beyond;

Reviewer: 2

Comments to the Author(s)
Review Report for RSOS-181686

General Comments:
This manuscript reported on the preparation and performance characterization of an electronic initiator which is based on nanothermite multilayer film and electrode plug. The ignition ability of electronic initiator with different thickness of energetic film was investigated and proved to be with an improved safety and reliability. The idea of integrating nanothermite multilayer film and electrode plug directly is interesting, simple and easy to implement. However, the reviewer has some concerns as stated in specific comments. These issues should be clarified before the manuscript could be considered for publication.

Specific Comments:
1. Section 3, the total thickness of nanothermite film was demonstrated to have little effect on heat release on page 5. However, page 9, authors stated the "heat output distinctly increased as the thickness raising", which seems to be a contradiction. Please explain. Besides, most papers show that the factor that affects the exothermic performance of multilayer energetic materials is the modulation period. But here, much better ignition ability in the gap test can be achieved by simply increasing the thickness of film. What's the possible mechanism? It would be better to add more in-depth discussion.
2. Introduction part, the authors used SCB as a comparative example. However, most of the cited references have no relation with SCB, e.g. [14], [17], [18], [20], [23-28] etc. Please check.
3. Figure 1, is it possible to add magnified figures of one electrode plug with and without nanothermite multilayer film, especially the area between electrodes, to see some details?
4. Section 2.2, the authors set the single layer thickness of Al film and CuO film were 100 nm and 200 nm, but in Figure 2a, the two layers seem not to be twice in thickness.

5. Figure 2b, why is there no obvious layering in EDS mapping? In addition, there are several unlabeled peaks in EDS plot.
6. Page 5, it seems the exothermic reaction after Al melting (660 C) contributes to heat release as well. Hence, the statement that "heat output was caused by solid-solid reaction" is not appropriate here.
7. Page 5, last sentence, the Figure SI 2 is voltage-current curve instead of DSC plots. Please check.
8. Page 6, the described Cu peaks (43.29, 50.43 and 74,13 degree) are not that obvious in the product curve of Figure 4. Besides, what do the sharp peaks in the product curve at around 53 and 55 degree stand for?
9. Page 7, please define function time. Because from Figure 6, the flame root of the 8 μ m-thick sample was extinguished earlier than that of 4.58 μ m-thick sample.
10. In order to highlight the advantages of Al/CuOX film, it is suggested to add the high-speed camera ignition images of traditional electronic plug (optional).
11. Page 9, "Although three kinds of initiators can easily fire the B-KNO3 tablet when it was intimately contacted with film, the ignition ability was much more differently." The authors did not provide related experimental data to support this sentence.
12. Page 9, last sentence, what does the "same condition" mean? Same contact or same gap distance?
13. Why the authors think the Al/CuOx layer can improve the safety of initiator? From this manuscript, the Al/ CuOx layer decreased the ignition voltage and it is possible to ignite even the gap increased to over 20mm. Doesn't it mean the initiator is less safe?
14. Two related papers should be included in the references: Journal of Materials Science 47, 1296-1305, 2012; ACS applied materials & interfaces 6, 10497-10505, 2014.
15. A carefully examine of the English grammar, sentence patterns and phrases of the entire manuscript sentence by sentence is sincerely recommended. E.g. the first sentence of Abstract part is completely unclear.

Author's Response to Decision Letter for (RSOS-181686.R0)

See Appendix A.

RSOS-181686.R1 (Revision)

Review form: Reviewer 1 (Yimin Chao)

Is the manuscript scientifically sound in its present form?
Yes

Are the interpretations and conclusions justified by the results?
Yes

Is the language acceptable?
Yes

Is it clear how to access all supporting data?
Yes

Do you have any ethical concerns with this paper?
No

Have you any concerns about statistical analyses in this paper?
No

Recommendation?
Accept as is

Comments to the Author(s)
The revision has addressed all points well.

Review form: Reviewer 2

Is the manuscript scientifically sound in its present form?
Yes

Are the interpretations and conclusions justified by the results?
Yes

Is the language acceptable?
Yes

Is it clear how to access all supporting data?
Yes

Do you have any ethical concerns with this paper?
No

Have you any concerns about statistical analyses in this paper?
No

Recommendation?
Accept with minor revision (please list in comments)

Comments to the Author(s)
Review Report for RSOS-181686.R1

General Comments:
The revised manuscript has been significantly improved, especially the introduction part. It can be considered for publication after the following minor issues being modified.

Specific Comments:
1. Please correctly mark the labels in Figure 1 and 7.
2. Page 6, is it better to move XRD result forward since there's no data from product?
3. Page 7, the authors are not sure about "the product Cu" according to last version of manuscript, and there's no evidence to prove Cu was eventually produced.
4. Please check the title of Figure SI 3.

Decision letter (RSOS-181686.R1)

01-Apr-2019

Dear Dr Ni:

On behalf of the Editors, I am pleased to inform you that your Manuscript RSOS-181686.R1 entitled "Gap initiation with 20.35 mm: an initiator integrating the Al/CuOx multilayer film and traditional electronic plug to enhance the ignition ability" has been accepted for publication in Royal Society Open Science subject to minor revision in accordance with the referee suggestions. Please find the referees' comments at the end of this email.

The reviewers and Subject Editor have recommended publication, but also suggest some minor revisions to your manuscript. Therefore, I invite you to respond to the comments and revise your manuscript.

• Ethics statement
If your study uses humans or animals please include details of the ethical approval received, including the name of the committee that granted approval. For human studies please also detail whether informed consent was obtained. For field studies on animals please include details of all permissions, licences and/or approvals granted to carry out the fieldwork.

• Data accessibility
It is a condition of publication that all supporting data are made available either as supplementary information or preferably in a suitable permanent repository. The data accessibility section should state where the article's supporting data can be accessed. This section should also include details, where possible of where to access other relevant research materials such as statistical tools, protocols, software etc can be accessed. If the data has been deposited in an external repository this section should list the database, accession number and link to the DOI for all data from the article that has been made publicly available. Data sets that have been deposited in an external repository and have a DOI should also be appropriately cited in the manuscript and included in the reference list.

If you wish to submit your supporting data or code to Dryad (http://datadryad.org/), or modify your current submission to dryad, please use the following link:
http://datadryad.org/submit?journalID=RSOS&manu=RSOS-181686.R1

• Competing interests
Please declare any financial or non-financial competing interests, or state that you have no competing interests.

• Authors’ contributions
All submissions, other than those with a single author, must include an Authors’ Contributions section which individually lists the specific contribution of each author. The list of Authors should meet all of the following criteria; 1) substantial contributions to conception and design, or acquisition of data, or analysis and interpretation of data; 2) drafting the article or revising it critically for important intellectual content; and 3) final approval of the version to be published.

All contributors who do not meet all of these criteria should be included in the acknowledgements.

We suggest the following format:

AB carried out the molecular lab work, participated in data analysis, carried out sequence alignments, participated in the design of the study and drafted the manuscript; CD carried out the statistical analyses; EF collected field data; GH conceived of the study, designed the study, coordinated the study and helped draft the manuscript. All authors gave final approval for publication.

• Acknowledgements
Please acknowledge anyone who contributed to the study but did not meet the authorship criteria.

• Funding statement
Please list the source of funding for each author.

Please note that we cannot publish your manuscript without these end statements included. We have included a screenshot example of the end statements for reference. If you feel that a given heading is not relevant to your paper, please nevertheless include the heading and explicitly state that it is not relevant to your work.

Because the schedule for publication is very tight, it is a condition of publication that you submit the revised version of your manuscript before 10-Apr-2019. Please note that the revision deadline will expire at 00.00am on this date. If you do not think you will be able to meet this date please let me know immediately.

To revise your manuscript, log into https://mc.manuscriptcentral.com/rsos and enter your Author Centre, where you will find your manuscript title listed under "Manuscripts with Decisions". Under "Actions," click on "Create a Revision." You will be unable to make your revisions on the originally submitted version of the manuscript. Instead, revise your manuscript and upload a new version through your Author Centre.

When submitting your revised manuscript, you will be able to respond to the comments made by the referees and upload a file "Response to Referees" in "Section 6 - File Upload". You can use this to document any changes you make to the original manuscript. In order to expedite the processing of the revised manuscript, please be as specific as possible in your response to the referees.

When uploading your revised files please make sure that you have:

1) A text file of the manuscript (tex, txt, rtf, docx or doc), references, tables (including captions) and figure captions. Do not upload a PDF as your "Main Document".
2) A separate electronic file of each figure (EPS or print-quality PDF preferred (either format should be produced directly from original creation package), or original software format)
3) Included a 100 word media summary of your paper when requested at submission. Please ensure you have entered correct contact details (email, institution and telephone) in your user account
4) Included the raw data to support the claims made in your paper. You can either include your data as electronic supplementary material or upload to a repository and include the relevant doi within your manuscript
5) All supplementary materials accompanying an accepted article will be treated as in their final form. Note that the Royal Society will neither edit nor typeset supplementary material and it will be hosted as provided. Please ensure that the supplementary material includes the paper details where possible (authors, article title, journal name).

Supplementary files will be published alongside the paper on the journal website and posted on

the online figshare repository (https://figshare.com). The heading and legend provided for each supplementary file during the submission process will be used to create the figshare page, so please ensure these are accurate and informative so that your files can be found in searches. Files on figshare will be made available approximately one week before the accompanying article so that the supplementary material can be attributed a unique DOI.

Once again, thank you for submitting your manuscript to Royal Society Open Science and I look forward to receiving your revision. If you have any questions at all, please do not hesitate to get in touch.

Kind regards,
Royal Society Open Science Editorial Office
Royal Society Open Science
openscience@royalsociety.org

on behalf of Professor R. Kerry Rowe (Subject Editor)
openscience@royalsociety.org

Reviewer comments to Author:
Reviewer: 2

Comments to the Author(s)
Review Report for RSOS-181686.R1

General Comments:
The revised manuscript has been significantly improved, especially the introduction part. It can be considered for publication after the following minor issues being modified.

Specific Comments:
1. Please correctly mark the labels in Figure 1 and 7.
2. Page 6, is it better to move XRD result forward since there's no data from product?
3. Page 7, the authors are not sure about "the product Cu" according to last version of manuscript, and there's no evidence to prove Cu was eventually produced.
4. Please check the title of Figure SI 3.

Reviewer: 1

Comments to the Author(s)
The revision has addressed all points well.

Author's Response to Decision Letter for (RSOS-181686.R1)

See Appendix B.

Decision letter (RSOS-181686.R2)

05-Apr-2019

Dear Dr Ni,

I am pleased to inform you that your manuscript entitled "Gap initiation with 20.35 mm: an initiator integrating the Al/CuOx multilayer film and traditional electronic plug to enhance the ignition ability" is now accepted for publication in Royal Society Open Science.

You can expect to receive a proof of your article in the near future. Please contact the editorial office (openscience_proofs@royalsociety.org and openscience@royalsociety.org) to let us know if you are likely to be away from e-mail contact. Due to rapid publication and an extremely tight schedule, if comments are not received, your paper may experience a delay in publication.

Royal Society Open Science operates under a continuous publication model (http://bit.ly/cpFAQ). Your article will be published straight into the next open issue and this will be the final version of the paper. As such, it can be cited immediately by other researchers. As the issue version of your paper will be the only version to be published I would advise you to check your proofs thoroughly as changes cannot be made once the paper is published.

On behalf of the Editors of Royal Society Open Science, we look forward to your continued contributions to the Journal.

Kind regards,
Andrew Dunn
Royal Society Open Science Editorial Office
Royal Society Open Science
openscience@royalsociety.org

on behalf of Prof R. Kerry Rowe (Subject Editor)
openscience@royalsociety.org

Follow Royal Society Publishing on Twitter: @RSocPublishing
Follow Royal Society Publishing on Facebook:
https://www.facebook.com/RoyalSocietyPublishing.FanPage/
Read Royal Society Publishing's blog: https://blogs.royalsociety.org/publishing/

Appendix A

Dear Editors and Reviewers,

Thank you for your letter and for the reviewers'comments concerning our manuscript entitled "Gap initiation with 20.35 mm: an initiator integrating the Al/CuOx multilayer film and traditional electronic plug to enhance the ignition ability" (ID RSOS-18168). Those comments are all valuable and very helpful for revising and improving our paper, as well as the important guiding significance to our researches. We have studied comments carefully and have made correction which we hope meet with approval. The main corrections in the paper and the responds to the reviewer's comments are as following.

Thanks for all the help.

Best wishes

Debin Ni

Reviewer: 1

1. replace the full stop with comma after plug in the second line in abstract;

Response:

The sentence was revised.

2. page 2, line 4 in the second paragraph, double check Al/Ni, it might be Al/NiO;

Response:

It should be Al/NiO, and Al/Ni was deleted.

3. page 5 line 7, replace weekly with weak;

Response:

The sentence of "and the low temperature weak exothermic peak was not expected to play a main role in the ignition process" was revised. The word of "weakly" was introduced to page 5 line 7, "One weakly exothermic peak"

4. page 5 line 16, the statement on thickness effect is incorrect;

Response:

As we known, the modulation period and modulation ratio will influence the heat release of nanothermite film. In this paper, three kinds of Al/CuOx multilayer film were the same modulation period and modulation ratio, the only difference was thickness. The heat release measured from DSC was the amount of heat of unit mass, it was no related to the thickness. In addition, the results of DSC were calculated by software, but the integral area of heat release was chosen manually, so the result will be different.

So, we think that the thickness of film has little effect on heat release.

5. page 6, line 4, replace was with were; a lot of grammar errors in this paragraph and beyond;

Response:

The grammar in this paragraph was double checked. And the English grammar, sentence patterns and phrases of entire manuscript sentences were carefully checked.

Reviewer: 2

1. Section 3, the total thickness of nanothermite film was demonstrated to have little effect on heat release on page 5. However, page 9, authors stated the “heat output distinctly increased as the thickness raising”, which seems to be a contradiction. Please explain. Besides, most papers show that the factor that affects the exothermic performance of multilayer energetic materials is the modulation period. But here, much better ignition ability in the gap test can be achieved by simply increasing the thickness of film. What’s the possible mechanism? It would be better to add more in-depth discussion.

Response:

I think that it is not contradictory. The heat release measured from DSC was the amount heat of unit mass, it is impacted on the modulation period and modulation ratio, and it is not related to the thickness and the mass of multilayered Al/CuOx film. On the other hand, it is a constant value when the structure of film is fixed. In page 9, the surface area of electrode plug is constant, and the amount heat of Al/CuOx multilayer film of unit mass is constant, the heat output of Al/CuOx multilayer film is only related to the quantity of Al/CuOx multilayer film. The quantity of film should be increased as the thickness raising, the total heat output should be increased as the thickness increasing.

2. Introduction part, the authors used SCB as a comparative example. However, most of the cited references have no relation with SCB, e.g. [14], [17], [18], [20], [23-28] etc. Please check.

Response:

These references were checked and deleted.

3. Figure 1, is it possible to add magnified figures of one electrode plug with and without nanothermite multilayer film, especially the area between electrodes, to see some details?

Response:

The images of electrode plug were magnified 100 times, and shown in the Figure 1.

4. Section 2.2, the authors set the single layer thickness of Al film and CuO film were 100 nm and 200 nm, but in Figure 2a, the two layers seem not to be twice in thickness.

Response:

The thickness of CuO and Al film were measured by the Nano Measurer software, and the results were shown in Table 1. The average thickness of CuO and Al film was 196.7 5nm and 101.18 nm, respectively.

Table 1 the measurement results of the thickness of CuO and Al film

right	thickness of CuO layer (nm)	Mean/nm	left	thickness of Al layer (nm)	Mean/nm
1	193.21	196.75	1	112.34	101.18
2	193.21		2	105.32	
3	199.65		3	101.87	
4	199.65		4	98.30	

5	186.77		5	94.79	
6	193.31		6	101.81	
7	193.21		7	108.83	
8	199.75		8	94.79	
9	212.52		9	101.81	
10	199.75		10	94.79	
11	193.21		11	98.30	

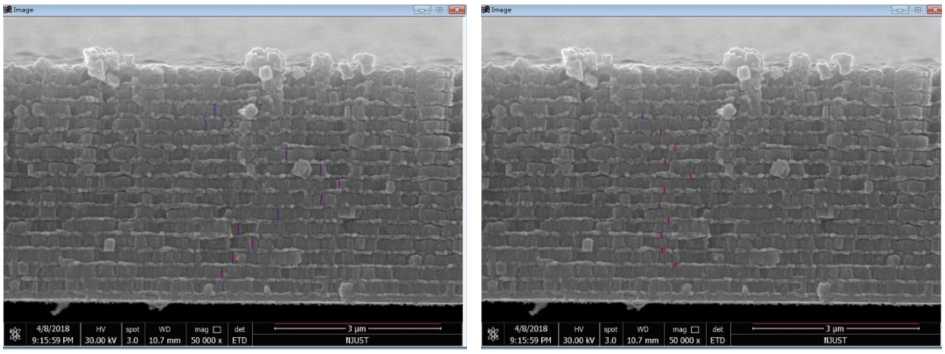

The measurement results of the thickness CuO（right） and Al film （left）

5. Figure 2b, why is there no obvious layering in EDS mapping? In addition, there are several unlabeled peaks in EDS plot.

Response:

The layer structure of EDS mapping is not obvious, it may be caused to the low resolution of SEM. The cross-section SEM image of Al/CuOx multilayer film was measured in Nanjing University of Science and Technology, the EDS of Al/CuOx multilayer film was measured in our lab.

The unlabeled peaks are Au and Si. Au is from the layer deposited onto samples prior to the SEM measurement in order to increase the electric conductivity, while Si signal is from the quartz substrate.

6. Page 5, it seems the exothermic reaction after Al melting (660 C) contributes to heat release as well. Hence, the statement that “heat output was caused by solid-solid reaction” is not appropriate here.

Response:

The sentence of “which was caused by the solid-solid reaction of Al with CuO” was deleted

7. Page 5, last sentence, the Figure SI 2 is voltage-current curve instead of DSC plots. Please check.

Response:

The DSC plots were replenished in the Figure SI 2.

8. Page 6, the described Cu peaks (43.29, 50.43 and 74.13 degree) are not that obvious in the product curve of Figure 4. Besides, what do the sharp peaks in the product curve at around 53 and 55 degree stand for?

Response:

Yes，the product of Cu peaks are not obvious in the Figure 4, it may be attribute to the low contents of Cu. It is hard to explain the peaks around 53 and 55 degree, although I have checked many papers, they may be caused by the contamination during the process of preparing the XRD sample. And I will continue to study what the peaks are and where they are come from.

I hope to delete the discussion about the production of multilayered Al/CuOx film，it is not the key of this paper.

9. Page 7, please define function time. Because from Figure 6, the flame root of the 8 μm-thick sample was extinguished earlier than that of 4.58 μm-thick sample.

Response:

The function time is derived from SCB devices, it is calculated from the voltage-current plots, the time that the current just pass the bridge wire is defined t_0, and the time that the current kept steady is defined t_1. the function time is the difference between t_1 and t_o , it is not observed by high-speed camera. (Peng Zhu, et al., Characterization of Al/CuO nanoenergetic multilayer films integrated with semiconductor bridge for initiator applications, Journal of Applied Physics, 2013, 113, 184505, doi: 10.1063/1.4804315)

10. In order to highlight the advantages of Al/CuO_X film, it is suggested to add the high-speed camera ignition images of traditional electronic plug (optional).

Response:

The high-speed camera ignition images of traditional electronic plug are shown in Figure SI 4.

11. Page 9, “Although three kinds of initiators can easily fire the B-KNO3 tablet when it was intimately contacted with film, the ignition ability was much more differently.” The authors did not provide related experimental data to support this sentence.

Response:

The test results of three kinds of initiators with different thickness of Al/CuO_X film were shown in Table S2.

12. Page 9, last sentence, what does the “same condition” mean? Same contact or same gap distance?

Response:

The same condition means that the electrode plug was contacted with B-KNO_3 tablet. The sentences of “The electrode plug without Al/CuOx multilayer film was also ignited under the same condition” were replaced by“The electrode plug without Al/CuOx multilayer film was tightly contacted to ignite the B-KNO_3 tablet”.

13. Why the authors think the Al/CuOx layer can improve the safety of initiator? From this manuscript, the Al/ CuOx layer decreased the ignition voltage and it is possible to ignite even the gap increased to over 20mm. Doesn’t it mean the initiator is less safe?

Response:

It does not mean the initiator is less safe, because the Al/CuOx layer could avoid the bridge wire contact with initiating explosive directly, especially for the extremely initiating explosive, such as LTNR, LA. The low ignition voltage of Al/CuOx layer is benefit to the micro-ignition system and

the gap initiation ability of Al/CuOx layer is promised to improve safety in the In—Line Ignition System.

14. Two related papers should be included in the references: Journal of Materials Science 47, 1296-1305, 2012; ACS applied materials & interfaces 6, 10497-10505, 2014.

Response:

The paper of “Journal of Materials Science 47, 1296-1305, 2012”was listed in the reference of 15. The paper of “ACS applied materials & interfaces 6, 10497-10505, 2014”was listed in the reference of 21.

15. A carefully examine of the English grammar, sentence patterns and phrases of the entire manuscript sentence by sentence is sincerely recommended. E.g. the first sentence of Abstract part is completely unclear.

Response:

The English grammar, sentence patterns and phrases of entire manuscript sentence were carefully checked.

Special thanks to you for your good comments

Appendix B

Dear Editors and Reviewers,

Thank you for your letter and for the reviewers'comments concerning our manuscript entitled "Gap initiation with 20.35 mm: an initiator integrating the Al/CuOx multilayer film and traditional electronic plug to enhance the ignition ability" (ID RSOS-18168). Those comments are all valuable and very helpful for revising and improving our paper, as well as the important guiding significance to our researches. We have studied comments carefully and have made correction which we hope meet with approval. The main corrections in the paper and the responds to the reviewer's comments are as following.

Thanks for all the help.

Best wishes

Comments to the Author(s)

Review Report for RSOS-181686.R1

General Comments:

The revised manuscript has been significantly improved, especially the introduction part. It can be considered for publication after the following minor issues being modified.

Specific Comments:

1. Please correctly mark the labels in Figure 1 and 7.

Response: the labels were checked.

2. Page 6, is it better to move XRD result forward since there's no data from product?

Response: It is not reasonable to move XRD result. So, we supplied the result in Paper. About 1 gram Al/CuOx multilayer film was ignited in an enclosed vessel, and the product of reaction was analyzed by XRD again, the plots of XRD were shown in Figure 4.

3. Page 7, the authors are not sure about "the product Cu" according to last version of manuscript, and there's no evidence to prove Cu was eventually produced.

Response: The plots of XRD were shown in Figure 4, and the results were shown that there was Cu in the product.

4. Please check the title of Figure SI 3.

Response: The title of Figure SI 3 was checked and corrected.

Reviewer: 1
Comments to the Author(s)
The revision has addressed all points well.

Special thanks to you for your good comments
